# Digital participatory workshops with patients and health professionals to develop an intervention for the management of polypharmacy: results from a mixed-methods evaluation and methodological conclusions

**DOI:** 10.1186/s40900-022-00387-1

**Published:** 2022-09-16

**Authors:** Jennifer Engler, Franziska Brosse, Truc Sophia Dinh, Astrid-Alexandra Klein, Maria-Sophie Brueckle, Jenny Petermann, Christiane Muth, Karola Mergenthal, Marjan van den Akker, Karen Voigt

**Affiliations:** 1 Institute of General Practice, Goethe-Universität Frankfurt Am Main, Theodor-Stern-Kai 7, 60590 Frankfurt, Germany; 2 Department of General Practice, Medical Clinic III, Faculty of Medicine Carl Gustav Carus, Technische Universität Dresden, Fetscherstraße 74, 01307 Dresden, Germany; 3 Department of General Practice and Family Medicine, Medical Faculty East-Westphalia, University of Bielefeld, Universitaetsstrasse 25, 33615 Bielefeld, Germany; 4 Department of Family Medicine, Care and Public Health Research Institute, Maastricht University, Maastricht, The Netherlands; 5 Department of Public Health and Primary Care, Academic Centre of General Practice, KU Leuven, Louvain, Belgium

**Keywords:** Patient participation, Stakeholder participation, Patient and public Involvement, Intervention development, Research design, Methods, Digital workshops, Polypharmacy, Intersectoral care

## Abstract

**Background:**

In the COVID-19 pandemic, numerous researchers postponed their patient and public involvement (PPI) activities. This was mainly due to assumptions on patients’ willingness and skills to participate digitally. In fact, digital PPI workshops differ from in-person meetings as some forms of non-verbal cues and body language may be missing and technical barriers may exist. Within our project HYPERION-TransCare we adapted our PPI workshop series for intervention development to a digital format and assessed whether these digital workshops were feasible for patients, health care professionals and researchers.

**Methods:**

We used a digital meeting tool that included communication via audio, video and chat. Discussions were documented simultaneously on a digital white board. Technical support was provided via phone and chat during the workshops and with a technical introduction workshop in advance. The workshop evaluation encompassed observation protocols, participants’ feedback via chat after each workshop on their chance to speak and the usability of the digital tools, and telephone interviews on patients’ and health professionals’ experiences after the end of the workshop series.

**Results:**

Observation protocols showed an active role of moderators in verbally encouraging every participant to get involved. Technical challenges occurred, but were in most cases immediately addressed and solved. Participants median rating of their chance to speak and the usability of the digital tool was “very good”. In the evaluation interviews participants reported a change of perspective and mutual understanding as a main benefit from the PPI workshops and described the atmosphere as inclusive and on equal footing. Benefits of the digital format such as overcoming geographical distance, saving time and combining workshop participation with professional or childcare obligations were reported. Technical support was stressed as a pre-condition for getting actively involved in digital PPI.

**Conclusions:**

Digital formats using different didactic and documentation techniques, accompanied by technical support, can foster active patient and public involvement. The advantages of digital PPI formats such as geographical flexibility and saving time for participants as well as the opportunity to prepare and hold workshops in geographically stretched research teams persists beyond the pandemic and may in some cases outweigh the advantages of in-person communication.

**Supplementary Information:**

The online version contains supplementary material available at 10.1186/s40900-022-00387-1.

## Plain English summary

Digital patient and public involvement (PPI) activities differ from in-person meetings. For example, some forms of non-verbal cues and body language are limited and technical barriers may exist. Therefore, some research teams were hesitant to switch to a digital PPI format during the COVID-19 pandemic and postponed their PPI activities.

In this paper, we aim to describe,

- how we adapted a PPI workshop series to a digital format,
- how patients and health care professionals experienced these digital workshops,
- and which conclusions we have drawn for future digital PPI activities.

The workshop evaluation encompassed workshop observation protocols, participants’ feedback via chat on their chance to speak and the feasibility of the digital tools, and telephone interviews on participants’ experiences.

The study results showed that moderators had an active role in verbally encouraging every participant to get involved. Technical challenges occurred, but were in most cases immediately addressed and solved. Most participants rated their chance to speak and the feasibility of the digital format as “very good”. They described the atmosphere as inclusive and on equal footing without hierarchy between different stakeholder groups. Participants reported benefits of the digital format such as overcoming geographical distance, saving time and combining workshop participation with professional or childcare obligations. They stressed technical support as a condition for getting actively involved in digital PPI.

We conclude that some advantages of digital PPI may persist beyond the pandemic. Therefore, we encourage research teams to discuss the question of digital or in-person PPI with the involved patients and health professionals and decide on a case-by-case basis.

**Supplementary Information:**

The online version contains supplementary material available at 10.1186/s40900-022-00387-1.

## Background

With the first wave of the COVID-19 pandemic in March 2020, the Health Research Authority UK (HRA) found that patient and public involvement (PPI) in the designing, managing or dissemination of research proposals submitted to the HRA dropped from 80% to only 20% [1]. A HRA organized workshop with public involvement facilitators from charities, National Health Services Trusts, regulators, universities, clinical research facilities and independent bodies identified partly false assumptions of researchers on the capacities and adaptiveness of PPI organizations and individuals as a main barrier [1]. According to the HRA, researchers believed that “public contributors would not be as motivated or available to contribute to research during an urgent public health crisis; public involvement groups would not be working because their usual ways of working had been drastically disrupted; there would not be enough time to carry out meaningful public involvement within study set-up timelines” [1]. While barriers such as a lack of technological hardware, software or personal skills did hinder virtual patient care and patient engagement in some cases [2, 3], other patient and public contributors adapted fast and had even more capacities when getting involved from home [1]. Furthermore, the virtual transformation of PPI due to the COVID-19 pandemic has eased other barriers such as financial and time-related costs for travel and accommodation and fostered transregional cooperation [3, 4]. Also work or childcare obligations can be combined more easily with digital PPI [3, 5] and digital PPI spaces may be more inclusive to people with accessibility requirements, as they allow to adapt temperature, volume and seating individually [3].

Therefore, also Lampa et al. referring to insights from observations of digital PPI meetings during the pandemic, suggest not to postpone PPI activities, but rather to reflect on the differences of in-person and digital formats and to adjust workshop planning accordingly [6]. By comparing observations from in-person PPI meetings with observations of digital PPI meetings with the same group, Lampa et al. conclude that in digital meetings communication is less spontaneous, as breaks are taken alone away from the screen. Furthermore, there is a lack of certain non-verbal cues in digital meetings such as turning one’s body towards a person or using eye-contact for communication. Therefore, it is also much harder to claim space in digital meetings, as these non-verbal cues cannot be used to attract the moderator’s attention: Everyone is sitting in front of his or her camera and seems to look at every other person directly. If someone speaks, he or she needs to speak to the whole group, which might also be a barrier. This limitation of the mentioned non-verbal cues is increased by digital tools that additionally diminish the room for faces on the screen such as white boards or screen-sharing. If not all participants can be shown beside a shared screen, some programs arrange participants in a way that favor persons who speak a lot, which may intensify gaps between confident and less confident participants [6]. To overcome these challenges, Lampa et al. conclude that the moderator must be aware of these challenges and take on a much more active and directive role than in in-person meetings to include all participants: The moderator can address participants directly, pose questions towards individual participants, structure discussions more actively and needs to set and supervise communication rules. The “raise hands” function is a tool that exists in digital meetings only, but may help to decrease speaking barriers, as it allows to make one’s wish to speak with only one click and creates a fair speaker list visible to everyone [6]. Furthermore, Lampa et al. recommend smaller meetings with fewer participants to support the inclusion of each participant’s view as well as decreasing technological barriers by guiding participants into the digital meeting room by providing instructions before the meeting, sending reminders and providing technical support [6]. However, when we started the planning of our digital participatory workshop series these reflections and advices were not published yet.

In our project HYPERION-TransCare (Heading to ContinuitY of Prescribing in EldeRly with MultImOrbidity iN Transitional Care), we aimed to develop an intervention for patients with polypharmacy (who use five or more medications) at the interface of outpatient and inpatient care in a co-design process [7, 8]. Several publications have highlighted the need and the benefits of the involvement of patients and health professionals in the development of complex interventions for randomized-controlled trials in health care [7, 9–11]. Additional to assessing evidence on the potential effectiveness of a complex intervention and making assumptions about target groups, outcomes, cost-effectiveness and intervention components, e.g. by systematic reviews, PPI in the development of complex interventions may decrease research waste by designing interventions that fit into the delivery context [9, 10]. Addressing relevance and feasibility from patients’ and other stakeholders’ perspectives and co-designing interventions may decrease future problems in the organisation of a RCT and the implementation of the intervention into real-world settings [7, 9–11]. Therefore, we planned a participatory workshop series from March to June 2021 involving lay patients (that is, patients who are not organized in a self-help group or within a professional patient organization), informal caregivers such as relatives or friends who provide care without payment or contract, patient representatives (that is, patients who are associated with a professional patient organization), health care assistants (HCAs) who support physicians in outpatient settings, e.g. in organizing patient flows, measuring patients’ blood pressure or blood removal, pharmacists, clinical information scientists and nurses and physicians from inpatient and outpatient settings. The overall aim of the workshop series was the development of a complex intervention supported by all stakeholder groups and included the adaptation and tailoring of intervention components and the discussion of implementation strategies and expected barriers and facilitators [8]. The relationship between patients, health care professionals and the research team throughout the participatory workshop series was considered a “partnership”, that is, all participants were involved in the decision-making process on intervention design [11].

Like many other research teams, we were forced to switch from the anticipated in-person format to a digital format because of the COVID-19 pandemic. On the one hand, this allowed us to pool our resources and to conduct workshops in a team of researchers from both sites (Dresden and Frankfurt) of our practice-based research network (PBRN) SaxoForN in Germany [12, 13], and encompassing patients and health professionals from both sites. On the other hand, the switch of the participatory workshop series to a digital format also raised some questions and concerns similar to those found by the HRA [1], especially as at that time few publications and little guidance on digital PPI activities existed. These questions guided the evaluation of our digital PPI workshop series:

- Which digital tools can we use to facilitate group discussions and to present, document and prioritize preliminary results within the PPI group?
- Will we be able to recruit enough patients and health professionals that respond positively to digital workshops and have the necessary technical skills? How can we best avoid barriers and foster inclusion?
- How can we facilitate familiarity and a trustful atmosphere in a digital workshop without coffee breaks and snacks for informal chats?
- How can we moderate workshops efficiently and how can we be responsive to all participants with limited in-person signals of body language and eye-contact? How can interaction within the group be facilitated in a digital format?

Today, some resources and methodological guides exist on concrete digital tools and how to use these in remote PPI activities [14–16]. Nevertheless, publications on evaluation data of digital PPI activities during the COVID-19 pandemic are rare, and most authors conclude that a sharing of researchers’, patients’ and health professionals’ experiences is crucial for a better understanding of which tools and formats work for which purpose and which participating group [1–4, 6]. This adds to considerations on the evaluation of PPI in general. Among others, Staley stresses that the impact and value of PPI is always context-specific [17]. The sharing of researchers’, patients’ and health care professionals’ experiences of PPI within a specific project as “knowledge in context” is therefore a valuable source for others to learn about the conditions, challenging and supporting factors of successful PPI in a specific context [17]. Sharing experiences on digital PPI within the pandemic is even more relevant when we regard digital PPI activities not only as a temporary substitute for in-person meetings, but reflect both on the advantages and challenges of digital PPI as a counterpart of in-person PPI activities in the future.

Following this principle, we aim to describe 1) how we put patient and health professional involvement in the development of an intervention into practice using a digital workshop format and 2) how patients and health care professionals experienced these participatory digital workshops and which methodological conclusions we can draw from the workshop evaluation.

## Methods

This paper follows the GRIPP2 reporting guideline [18]. We asked patient participants for contribution in authorship, but due to time constraints and (English) language barriers this was not possible. The manuscript and the plain language summary were language checked by a native speaker with no background in medicine or health services research.

### Participatory digital workshops

#### The HYPERION-TransCare study

In this paper, we refer to results of the evaluation of digital participatory workshops with patients with polypharmacy, their informal caregivers, patient representatives and health professionals from inpatient and outpatient settings who care for this patient group. The workshops were part of the project HYPERION-TransCare that aimed to co-design an intervention for patients with polypharmacy at the intersection of outpatient and inpatient care [8]. Older patients with multiple diseases and multiple medications have complex care needs. Information continuity between outpatient and inpatient care is very important for this group to prevent medical errors, inappropriate treatment, patient concerns and a lack of confidence in healthcare. Therefore, HYPERION-TransCare aimed to develop an intervention to improve informational continuity of care at the interface between general practice and hospital care [8].

HYPERION-TransCare was conducted in Germany at two sites of the PBRN SaxoForN [1, 2]: the Department of Family Medicine Dresden and the Institute of General Practice Frankfurt am Main. Prior to the workshops, we conducted interviews with patients and health professionals to get an overview of experiences, problems and possible solutions at the interface of outpatient and inpatient care for patients with polypharmacy. Based on the results of the interviews we developed contents and methods for the workshops. The co-designed intervention developed in the workshops will be tested in a following pilot study.

#### Recruitment for digital workshops

To recruit participants for the digital PPI workshops, we asked participants of the prior interviews whether they aimed to join in the participatory workshop series as well. For the initial recruitment of interview participants, general practitioners (GPs) and HCAs were recruited via the PBRN SaxoForN. All other professions were recruited using purposive sampling and from multiple hospitals, pharmacies, and care services. Patients and informal caregivers were recruited via general practices and announcements in local papers. An appointed patient representative from the Federal Joint Committee (“Gemeinsamer Bundesausschuss”)—who is involved in discussions on the selection of health care services to be covered by the public health insurance in Germany—represented the broader views of patients.

We asked 25 participants from the prior interview series to participate in the participatory workshops. Nine were interested, but patients refused to participate because of the digital workshop format and professionals refused to participate because of pandemic-related increasing workload. 16 participants from the prior interviews actually participated in one or more digital workshop. Based on existing professional networks of the project staff, further participants (n = 14) of the workshops were recruited by personal invitations. Finally, 30 persons participated at least in one digital workshop.

#### Digital Workshop design

The preparation of the research team encompassed a training course in plain language and a workshop on conducting digital PPI workshops and prioritization techniques within digital meetings. We planned a participatory workshop series that consisted of five intensive workshops (IWS) with rather homogeneous stakeholder groups and two synthesis workshops (SWS) that involved stakeholders from all groups (see Table 1).

**Table 1:** Structure and participants of the participatory workshop series

Workshop	Date	Aims and content	Participants (per stakeholder group)
Technical introduction workshop	29.03.2021	Getting to know stakeholders and the research team Getting to know and trying out the functions of BBB Breaking down technical barriers for workshop participation Building trust in the research team and in one’s own technical skills Getting to know the structure, aim and content of the following workshops Closing: summary, feedback, forecast	Patients, patient representatives and informal caregivers
Intensive Workshop 1	30.30.2021	Getting to know stakeholders and the research team Presentation of prior study results on challenges of information continuity between outpatient and inpatient care Adding additional challenges from stakeholders’ perspectives and prioritizing challenges Collecting possible solutions from stakeholders’ perspective and prioritizing possible solutions Closing: summary, feedback, forecast	Patients, patient representatives and informal caregivers
Intensive Workshop 2	07.04.2021	Getting to know stakeholders and the research team Presentation of prior study results on challenges of information continuity between outpatient and inpatient care Adding additional challenges from stakeholders’ perspectives and prioritizing challenges Collecting possible solutions from stakeholders’ perspectives and prioritizing possible solutions Closing: summary, feedback, forecast	Health care assistants, inpatient and outpatient nurses
Intensive Workshop 3	05.05.2021	Getting to know stakeholders and the research team Reflecting upon IWS 1 and IWS 2 Presenting the results from IWS 1 and IWS 2 Input on a digital nationwide medication plan („Bundeseinheitlicher Medikationsplan “) and discharge management Discussing possible solutions including feasibility Closing: summary, feedback, forecast	Patients, patient representatives and informal caregivers, health care assistants, inpatient and outpatient nurses
Intensive Workshop 4	28.04.2021	Getting to know stakeholders and the research team Presentation of prior study results on challenges of information continuity between outpatient and inpatient care Adding additional challenges from stakeholders’ perspectives and prioritizing challenges Collecting possible solutions from stakeholders’ perspective and prioritizing possible solutions Closing: summary, feedback, forecast	Clinical doctors, clinical pharmacists, clinical information scientists
Intensive Workshop 5	05.05.2021	Getting to know stakeholders and the research team Reflecting upon the last workshop Presenting the results from IWS 4 Input on a digital nationwide medication plan („Bundeseinheitlicher Medikationsplan “) and discharge management Discussing possible solutions including feasibility Closing: summary, feedback, forecast	Clinical doctors, clinical pharmacists, clinical information scientists
Synthesis Workshop 1	16.06.2021	Getting to know stakeholders and the research team Presenting the results from IWS 1–5 Discussing characteristics of the intervention in subgroups Presenting and consenting on characteristics of the intervention (all participants) Closing: summary, feedback, forecast	All stakeholders
Synthesis Workshop 2	30.06.2021	Presenting the results of SWS 1 Presenting a preliminary intervention design Discussing and finalizing the preliminary intervention design in subgroups Presenting discussion results from subgroups to all participants Discussing possible endpoints, feasibility and implementation of the intervention Closing: summary, feedback, forecast	All stakeholders

The goal of the IWSs was (1) to assess a shared impression of problems as well as a prioritization of problems and (2) to collect possible solutions and components of a medication information management intervention for transitional care and discuss their estimated appropriateness and feasibility. We involved rather homogeneous stakeholder groups in the IWSs to reduce speaking barriers, facilitate group discussions on equal footing and to focus on stakeholder-specific topics. As a starting point of the first IWS, results from the individual in-depth interviews were briefly presented. In each subsequent workshop; the topics were based on the results of previous workshops, resulting in an iterative development of the complex intervention.

The results of the IWSs were provided and discussed in two SWSs with participants from all stakeholder groups. We aimed to include at least two representatives of each stakeholder group per SWS. The SWSs aimed at the development of a complex intervention supported by all stakeholder groups and included the adaptation and tailoring of intervention components. Furthermore, implementation strategies and expected barriers and facilitators were mapped.

Prior to the workshop series, participants from the patient group were offered a technical introduction workshop with the aim of making them familiar with the technical tools and methods of the open source web conferencing system BigBlueButton (BBB) [19] that we planned to use in the digital workshops. We favored BBB over Zoom and GoTo Meeting, because our institutions recommended using BBB for reasons of data security (data is stored on servers within Germany only). Furthermore, different from Zoom or GoTo Meeting, users were not asked to download an app. The BBB version that we could access through our institution for free provided the possibility for breakout sessions and additional interactive tools, and some members of our team were experienced with BBB because we had used it before for digital teaching, digital continuous education, information events and meetings with GPs and HCAs within our PBRN before. All participants were informed in advance that we offer a financial reimbursement for their contributions in the design of our intervention. Reimbursement was not calculated based on an hourly rate but paid as a lump sum and as a symbolic appreciation: 100 euros per workshop and 50 euros per telephone interview for evaluation. The contribution of all participants from all stakeholder groups were equally appreciated and equally necessary to design a reasonable and feasible intervention, therefore all participants were equally reimbursed.

Within the technical introduction workshop, we practiced features such as raising one’s hand, using the chat function, muting and unmuting the microphone, writing or stamping on the shared whiteboard and answering BBB-surveys. Furthermore, this first coming-together of patients, patient representatives and informal caregivers was meant as a group-building activity: to make them familiar with each other and the researchers, to build trust and self-confidence, and to train raising one’s voice. We expected a need of technical training in the patient group, because we assumed that digital group formats were barely part of their daily lives. By contrast, we expected more digital skills based on current work tasks in the group of health care professionals consisting of clinical doctors, pharmacists, clinical information scientists, HCAs and nurses from inpatient and outpatient settings.

Each workshop was moderated by an experienced moderator with a background in health care research or medical education, but with no direct involvement in the HYPERION-TransCare project. Moderators were briefed by two researchers on the overall study aim, results and discussions from prior workshops, and the planned agenda of each workshop, including a time plan, leading questions and discussion topics. Moderators were asked to pay special attention to the involvement of all participants.

Apart from the moderator, each workshop was accompanied by at least four members from the study team: one for technical support, one writing a detailed protocol for intervention development, one taking notes on the open online whiteboard Miro [20] and one writing observation protocols on communication for workshop evaluation. For in-depth discussions we divided the SWS in smaller subgroups who were transferred to a separate private digital meeting room. We documented discussions on PowerPoint slides on a shared screen together with participants.

All participants received written study materials and signed an informed consent form prior to workshop participation. Material was sent by post or mail and included information on the course, time and effort of the workshop series, information on data security and the consent form. After returning the consent form, participants were sent an invitation letter, a short introduction to BBB and a form to fill in for financial reimbursement with a stamped addressed envelope by mail. Furthermore, to foster familiarity and substitute the informal amenities of in-person workshops, we sent care packages as incentives in preparation for the first IWS including snacks, drinks and writing material. The access link and a reminder were sent shortly before each workshop. The workshops took place between March and June 2021.

### Evaluation

To assess the feasibility of PPI within our digital workshop format and participants’ experiences, we used a mixed-methods approach including three evaluation methods: 1) workshop observation protocols that focused on communication; 2) participants’ rating via chat after each workshop on their chance to speak and the usability of the digital format; 3) telephone interviews with participants from each stakeholder group.

#### Workshop observation protocols

We wrote an observation protocol per workshop that focused on communication. These communication protocols were guided by three themes and questions:

1. Activation: How are workshop participants activated by the moderator to speak up and share their experiences and opinions?
2. Interaction: How do workshop participants interact with each other? Are they responsive to each other? How is interaction facilitated by the workshop moderator?
3. Challenges: Which challenging situations occur and how are they solved or not solved?

Protocols were written continuously during digital workshops by a member of the research team trained in qualitative methods. After the workshop series was completed, each protocol was analyzed by JE with regard to the guiding questions. In each protocol, passages that gave answers to Question 1, 2 or 3 were marked in a different color. These passages were analyzed per color/question and synthesized in a final document that summarized findings per question across workshops.

#### Participants’ feedback via chat

After each workshop, we asked all participants to rate on a 6-point-rating scale how well they got a chance to speak (1 = “very good”, 2 = “good”, 3 = “satisfactory” 4 = “sufficient”, 5 = “poor”, 6 = “deficient”) and how well they got along with the digital format and video conference system (1 = “very good”, 2 = “good”, 3 = “satisfactory” 4 = “sufficient”, 5 = “poor”, 6 = “deficient”) and to send this rating to a named researcher participating in the workshop in a private chat message. Data was transferred into an Excel sheet and analyzed with regard to median, minimum and maximum per group (“patient group” = patients, patient representatives, informal caregivers; “health care professional group” = HCAs, nurses and physicians from inpatient and outpatient settings, pharmacists, medical information scientists) and per workshop. Calculations are based on valid answers only.

#### Telephone interviews

After the workshops series was completed, we informed all participants via email that we aimed to contact them for an evaluation interview via phone and asked them to let us know, if they did not wish to be contacted. We used a purposive sampling approach [21] with the aim to equally include participants from the patient group and the health care professional group (including participants from inpatient and outpatient settings encompassing physicians, nursing and HCAs). Furthermore, we aimed to conduct interviews within four weeks after the completion of the workshop series. We developed an interview guide for an interview length of maximum 15 min that focused on the following aspects: motivation and concerns to participate, interaction and collaboration between workshop participants in the workshop including challenges and especially positive or negative situations, perceived influence on the development of the intervention and the digital workshop format. The interview guide is provided as Additional file 1. Interviews were audiotaped and analyzed with thematic analysis [22] in accordance with the above mentioned pre-defined categories of the interview guide by JE. Selected passages were paraphrased or transcribed verbatim and put into an excel sheet with the categories. We analyzed the segments with regard to recurring themes and experiences as well as marginal standpoints.

## Results

### Participants

Overall, 30 patients and health professionals participated in our workshop series with a median of 10 participants per technical introduction, IWS or SWS (min: 6; max: 13). The technical introduction workshop was visited by six participants from the patient group. One patient was unable to turn on his laptop camera and microphone during the technical introduction workshop and therefore dropped out. We conducted telephone interviews for evaluation with eight participants. Six of the eight telephone interview participants (two from the patient group and four from the health professional group) participated in at least one IWS and one SWS, whereas two participants from the patient group participated in an IWS only. All participants’ characteristics per workshop and evaluation interview can be found in Table 2.

**Table 2:** Description of participants per workshop and evaluation interviews

			Overall study participation (N = 30)	Technical introduction workshop (N = 6)	IWS 1 (N = 7)	IWS 2 (N = 9)	IWS 3 (N = 10)	IWS 4 (N = 12)	IWS 5 (N = 9)	SWS 1 (N = 13)	SWS 2 (N = 13)	Evaluation interviews (N = 8)
Gender		Male (N%, n)	43%, n = 13	67%, n = 4	57%, n = 4	11%, n = 1	40%, n = 4	58%, n = 7	67%, n = 6	46%, n = 6	38%, n = 5	25%, n = 2
		Female (N%, n)	57%, n = 17	33%, n = 2	43%, n = 3	89%, n = 7	60%, n = 6	42%, n = 5	33%, n = 3	54%, n = 7	62%, n = 8	75%, n = 6
		other (N%, n)	0	0	0	0	0	0	0	0	0	0
Age		30–40 years (N%, n)	23%, n = 7	0	0	33%, n = 3	30%, n = 3	25%, n = 3	33%, n = 3	23%, n = 3	31%, n = 4	0
		41–50 years (N%, n)	30%, n = 9	0	0	44%, n = 4	30%, n = 3	50%, n = 6	44%, n = 4	38%, n = 5	38%, n = 5	38%, n = 3
		51–60 years (N%, n)	27%, n = 8	17%, n = 1	29%, n = 2	22%, n = 2	20%, n = 2	25%, n = 3	22%, n = 2	31%, n = 4	23%, n = 3	25%, n = 2
		61 years or older (N%, n)	20%, n = 6	83%, n = 5	71%, n = 5	0	20%, n = 2	0	0	8%, n = 1	8%, n = 1	38%, n = 3
Stakeholder group	Patients	Patient (N%, n)	20%, n = 6	83%, n = 5	71%, n = 5	n.a	20%, n = 2	n.a	n.a	8%, n = 1	8%, n = 1	38%, n = 3
		Patient representative (N%, n)	3%, n = 1	0	14%, n = 1	n.a	10%, n = 1	n.a	n.a	8%, n = 1	8%, n = 1	0
		Informal caregiver (N%, n)	3%, n = 1	17%, n = 1	14%, n = 1	n.a	10%, n = 1	n.a	n.a	8%, n = 1	0	13%, n = 1
		Overall patient group (N%, n)	27%, n = 8	100%, n = 6	100%, n = 7	n.a	40%, n = 4	n.a	n.a	23%, n = 3	15%, n = 2	50%, n = 4
	Health care professionals	Health care assistant (N%, n)	13%, n = 4	0	n.a	56%, n = 5	30%, n = 3	n.a	n.a	8%, n = 1	15%, n = 2	13%, n = 1
		Inpatient nurse (N%, n)	7%, n = 2	0	n.a	22%, n = 2	20%, n = 2	n.a	n.a	0	8%, n = 1	0
		Outpatient nurse (N%, n)	10%, n = 3	0	n.a	22%, n = 2	10%, n = 1	n.a	n.a	23%, n = 3	15%, n = 2	13%, n = 1
		Inpatient physician (N%, n)	13%, n = 4	0	n.a	n.a	n.a	33%, n = 4	33%, n = 3	23%, n = 3	8%, n = 1	13%, n = 1
		Outpatient physician (N%, n)	17%, n = 5	0	n.a	n.a	n.a	33%, n = 4	22%, n = 2	8%, n = 1	23%, n = 3	13%, n = 1
		Pharmacist (N%, n)	3%, n = 1	0	n.a	n.a	n.a	8%, n = 1	11%, n = 1	0	0	0
		Clinical information scientist (N%, n)	10%, n = 3	0	n.a	n.a	n.a	25%, n = 3	33%, n = 3	15%, n = 2	15%, n = 2	0
		Overall health care professionals group (N%, n)	73%, n = 22	0	n.a	100%, n = 9	60%, n = 6	100%, n = 12	100%, n = 9	77%, n = 10	85%, n = 11	50%, n = 4

*IWS* Intensive Workshop; *SWS* Synthesis Workshop

### Observation protocols

Generally, each workshop was conducted by an organizing team of researchers (8–12 persons) that provided technical support, wrote protocols for intervention development or observation protocols with regard to communication. At least two members presented data and documented discussions and results of prioritization processes on a digital whiteboard. Each workshop was held by an external moderator who was not part of the study team. In the SWSs even more moderators were present (one for each subgroup discussion). The video conference system BigBlueButton [19] fades out participants with turned off camera (instead of e.g. showing a black screen with the participant’s name). Therefore, the high number of researchers in the background were not visible and the audiovisual focus was on the workshop participants. However, all present members of the research team were introduced at the beginning of the workshop by name and video, before they switched off the camera.

#### Activation

At the beginning of each workshop all participants were welcomed by name when entering the digital meeting room. This was also the case when somebody entered later during the workshop: He or she was welcomed and got a short introduction about the status quo. At the beginning of each workshop, the moderator gave a short technical introduction to the virtual meeting, e.g. how to switch on/off the microphone and the camera, how to use the chat function and how to virtually raise one’s hand. Participants were asked to switch on their camera and to either use the chat function or virtually raise their hands in case they wanted to make a contribution. However, moderators encouraged participants to speak freely, in case the moderator did not react to chat or hand-raising or participants could not use these functions. Moderators stressed the confidentiality of everything that was shared during workshops. At the start of the IWSs, all participants were asked to introduce themselves by naming the group they represented and by sharing a situation that represents a typical problem in the medication management of patients with polypharmacy at the intersection of outpatient and inpatient care. This facilitated both a clear role description and a common ground for further discussions, as all participants started with a personal experience (instead of exchanging abstract knowledge). Moderators described their roles as “facilitating and structuring the discussion” and research team members as “observing, listening and documenting your ideas”. During the workshops, moderators consistently asked individual participants by name to share their experiences and opinions, whether they agreed or disagreed or whether they aimed to add something to the remarks of other participants or the documentation on the virtual white board.

#### Interaction

Especially in the IWSs, participants reacted to each other’s contributions and initiated conversations. In the SWSs, discussions between participants were rare and moderators more actively encouraged participants to react to each other’s contributions and to add their opinion. This was facilitated by the ongoing documentation on the virtual whiteboard. Short conversations rather occurred between moderators and participants. However, in the feedback round at the end of the SWSs, participants emphasized the value of other perspectives and felt that they learnt something new.

#### Challenges and solutions

During all workshops technical challenges occurred: Participants dropped out of the meeting and had problems to re-enter, they were not able to switch on their camera or microphone or to use the chat function for comments. These challenges were promptly solved by the technical support via phone: they contacted participants and supported them one-on-one, so that no one dropped out unnoticed. Furthermore, moderators encouraged participants with technical troubles to use an alternative communication channel (chat, phone).

For some participants the figures with study results were too small and therefore difficult to understand. In these cases, the moderators explained how to enlarge the display and made certain that the problem was solved before continuing. During the IWSs some participants did not clearly understand how the prioritization process was working, what their part in this was and which steps the moderators and the research team conducted on the virtual white board. Instructions were recapitulated and paraphrased by the moderators.

The observing researchers felt that these efforts to include every participant despite technical challenges facilitated the feeling that every participant’s contribution was valuable and useful.

### Participant feedback via chat

Most participants of the patient group and participants of the health professional group rated their chance to speak during the workshops as “very good”. This applies both to the IWSs in homogeneous groups and the SWSs where members from all groups came together (see Table 3).

**Table 3:** Participants' feedback via chat on their chance to speak and usability of the digital format

	Patient group	Health care professional group	All participants
Chance to speak (median [min; max], n, valid n)			
IWS1	1 [1; 2], n = 7, valid n = 6	n.a	1 [1; 2], n = 7, valid n = 6
IWS2	n.a	1 [1; 1], n = 9, valid n = 9	1 [1; 1], n = 9, valid n = 9
IWS3	1 [1; 1], n = 4, valid n = 4	1 [1; 1], n = 6, valid n = 6	1 [1; 1], n = 10, vaid n = 10
IWS4	n.a	1 [1; 3], n = 12; valid n = 11	1 [1; 3], n = 12; valid n = 11
IWS5	n.a	1 [1; 1], n = 9; valid n = 7	1 [1; 1], n = 9; valid n = 7
SWS1	1 [1; 1], n = 3; valid n = 2	1 [1; 2], n = 10; valid n = 6	1 [1; 2], n = 13; valid n = 8
SWS2	Group assignment missing	1 [1; 5], n = 13; valid n = 7	
Overall	1 [1; 2], n = 14, valid n = 12	1 [1; 3], n = 46; valid n = 39	1 [1; 5], n = 73; valid n = 58
Usability of the digital workshop (median [min; max], n, valid n)			
IWS1	1 [1; 2], n = 7, valid n = 6	n.a	1 [1; 2], n = 7, valid n = 6
IWS2	n.a	1 [1; 4], n = 9, valid n = 9	1 [1; 4], n = 9, valid n = 9
IWS3	1 [1; 1], n = 4, valid n = 4	1 [1; 2], n = 6, valid n = 6	1 [1; 2], n = 10
IWS4	n.a	2 [1; 4], n = 12, valid n = 11	2 [1; 4], n = 12, valid n = 11
IWS5	n.a	2 [1; 2,5], n = 9, valid n = 7	2 [1; 2,5], n = 9, valid n = 7
SWS1	1 [1; 1], n = 3, valid n = 2	1 [1; 3], n = 10, valid n = 6	1 [1; 3], n = 13, valid n = 8
SWS2	Group assignment missing	2 [1; 5], n = 13, valid n = 7	
Overall	1 [1; 2], n = 14, valid n = 12	1 [1; 4], n = 46, valid n = 39	1 [1; 5], n = 73, valid n = 58

Answers to the questions "Did you get a chance to speak today?" and "How did you get along with the digital format and video conference system today?" on a 6-point-rating scale (1 = “very good”, 2 = “good”, 3 = “satisfactory” 4 = “sufficient”, 5 = "poor", 6 = "deficient"), grouped by participants from the patient group (patients, patient representatives, informal caregivers) and participants from the health care professional group (health care assistants, nurses and physicians from inpatient and outpatient settings, pharmacists, medical information scientists), median with minimum and maximum rating per group and overall participants based on valid n; IWS = Intensive Workshop, SWS = Synthesis Workshop

Participants from the patient group assessed that they got along with the digital format and video conference system “very well” across all workshops, whereas the median rating in the health professional group for some workshops was only “good”, with maximum ratings up to “poor”. Two participants of the health professional group reported a non-working camera and poor internet connection in relation with their rating. These circumstances lay outside of our reach as workshop coordinators.

### Telephone interviews

Telephone interviews for workshop evaluation took place at the beginning of July 2021. The median duration of the telephone interviews was 11 min, ranging from 8 to 12 min.

#### Motivation to participate and concerns in advance

Participants from all groups mentioned their wish to make a difference and contribute to an improvement of health care by sharing their experiences. Many reported also that they were curious to “*take a look behind the scenes*” (patient) and to get to know other perspectives on a shared problem of everyday patient care. Two participants from the health professional group mentioned that they had to participate in research to get an accreditation as research practice within our PBRN and that the HYPERION-TransCare project was the next available project for this purpose.

Most participants mentioned no concerns in advance. Especially participants from the patient group reported that they “*fully trusted*” (patient) the research team and that they relied on prior positive experiences with the research team:

“Everything’s ok as long as not too much professional language is bandied about and that was not the case” (patient).

A participant from the health professional group mentioned that she had questioned the feasibility of a participatory workshop involving multiple stakeholder groups:

“How should that work, when so many people are sitting at so many different positions, how should that work as a real workshop?” (inpatient physician).

#### Interaction within the workshops: Facilitating a change of perspective and exchange of experiences

When asked to describe the atmosphere within the workshop and the interaction between participants, all interviewees stressed that the workshop allowed them a change of perspective and that they got to know the experiences of other stakeholders:

“It was interesting for me to get to know all these different opinions and perspectives” (patient).

“It was great! Especially the client, it was great that he took part and shared his experiences. The mix was amazing: Some general practitioners participated and health care assistants, their perspective is well-known to us, but what inpatient nurses experience – we just didn’t know so far” (outpatient nurse).

“What I experienced positively and what I appreciate a lot are these multiple perspectives; that you get to know the views of others that are working on the same problem” (HCA).

The mix of patients and health professionals from inpatient and outpatient settings was described as “*interesting, very broadly based, wide-ranging*” and “*communicative, open, keen on debate”* (inpatient physician).

Health care professionals primarily stressed the benefits of getting to know perspectives from other settings, whereas patient participants also stressed the exchange of experiences within the patient group in a sense of peer support as highly beneficial. A patient participant wished for more time to exchange individual experiences amongst patients next time, and another patient reported a feeling of endorsement when she heard about another participants’ experiences in hospital:

“A man needed to go into the hospital several times and he reported so many drawbacks. For me this was enriching, to hear that this was disagreeable not only for me” (patient).

She wished that contact information was shared amongst participants so that they could stay in contact also after the end of the study.

#### Interaction within the workshops: transparent communication on equal footing

Another important aspect that participants mentioned when asked about the atmosphere and interactions in the workshop was the perceived equality of all participants:

“For me the atmosphere was efficient and I felt good. […] In my opinion, there was no discrepancy, everyone was on equal footing so to speak” (informal caregiver).

“Everyone was equal, everyone was able to have their say” (inpatient physician).

Especially for patient participants, the technical introduction workshop was an important precondition to feel incorporated and make their contributions:

“It started with an introduction workshop to the technical tools that they planned to use. Often the problem is: You want to, but you simply cannot do it, because you are not experienced with the technicalities. I have nothing to complain about” (informal caregiver).

Furthermore, the transparent documentation of discussions and results during the workshops visible for everyone on the digital whiteboard facilitated a feeling of participation and co-production:

“I liked that everything was written done simultaneously. One person of the team always documented everything and assembled the results […] Amazing how things developed during the workshop, I liked that very much” (inpatient physician).

Furthermore, some participants wished for an event involving all participants and researchers for the presentation of the final results.

#### Perceived influence on intervention development and perceived challenges

All participants reported that they felt that they were able to make a contribution within the workshops and were able to influence the development of the intervention:

“I was able to tell what I have experienced and what I recommended was taken up, awesome” (outpatient nurse).

“There were several things that weren’t considered by physicians and that all came on the list immediately, not bad!” (patient).

Nevertheless, several participants from both groups doubted the successful implementation of the intervention and raised concerns with regard to health care structures:

“I can hardly imagine that this can be implemented, something really groundbreaking, but I’d be happy if you succeed” (inpatient physician).

“I am lacking faith that you’ll be able to carry your point” (informal caregiver).

Two participants from the health professional group felt uncomfortable with the openness and the iterative development of the intervention within the workshops, and the final intervention design did not fully meet their priorities:

“We weren’t sure what the content is, what the goals are and what’s the outcome at the end. That was a bit obscure at the beginning, and it didn’t fit our expectations. But we went through with it” (outpatient physician).

A patient participant mentioned that she had wished for more information in advance so that she could have better prepared herself for the questions raised in the workshop.

#### Digital format: geographical flexibility and saving time

Participants from all groups stressed the advantages of saving time and geographical flexibility that digital workshops provide. Participants furthermore positively stressed the possibility of getting to know colleagues from across the country or to combine child care and workshop participation:

“I was flexible that way. Driving somewhere – I couldn’t have managed that, because I have no one for my children. But so, the children were downstairs and I was able to enjoy my workshop upstairs” (outpatient nurse).

Another participant wished for different workshop hours next time, as she felt that the intersection of afternoon and early evening should be reserved for active family time. Most participants mentioned that they wished that digital formats persisted beyond the pandemic and stressed that “*personal encounters were possible*” (informal caregiver) also in the participatory digital format.

#### Digital format: prior experiences and the importance of technical support

Most participants reported that—in the second year of the pandemic—experiences and prior knowledge with video conference systems and digital formats existed both in the professional area, e.g. digital team meetings or online education, and in private settings, e.g. throughby attending digital language courses. However, even a patient participant with minor experience stressed that it was not challenging for him to participate in the digital workshops. He stressed that the digital format was *“well-arranged and very easy, even for me who isn’t sitting at the computer that often”* (patient). Another patient participant in this sense stressed the importance of the technical introduction workshop, which he felt was “*very helpful*” (informal caregiver) and in his opinion a precondition for digital participatory workshops.

## Discussion

Workshop observation protocols on communication, participants’ feedback via chat and telephone interviews with participants showed that participatory digital workshops for intervention development were feasible both for researchers, patients and health care professionals.

By sharing our “knowledge in context” [17] on the development and evaluation of digital PPI, we aim to inform discussions on the challenges and advantages of digital formats in PPI also beyond the pandemic and support other research teams in planning their PPI workshops digitally.

### The role of the research team in digital PPI workshops

The switch to a digital format, i.e. the selection and acquisition of digital tools and the adaption to the workshop goals, initially required a lot of resources on the research team’s side. However, by pooling resources across locations, we were able to work in a larger team and to share tasks across sites. Therefore, additional tasks in the organization of digital PPI workshops such as keeping an eye on the chat and providing technical support to guide participants into the meeting and help them with technical troubles during the meeting could be managed by the enhanced organizing team. These extra tasks were necessary and totally paid off, as evaluation interviews showed that participants appreciated this technical support a lot and that it gave them a feeling of safety. The saving of expenses for accommodation and catering could be used to support (digital) workshop features and the acquisition of workshop moderators from our network across the country.

Similar to the findings of Lampa et al. [6], our workshop observation protocols show that workshop moderators had an essential role in activating workshop participants by welcoming participants by name and giving status quo instructions to participants that arrive late or rejoin the meeting, by monitoring the digital “raised hands” list, the chat and participants who signal their will to speak in other ways, by actively asking individual (silent) participants for their opinion and by being patient and inclusive in cases of technical troubles. Due to the limitation of some forms of body language and non-verbal cues, the importance of verbal communication and good moderation and communication skills are even more important in digital PPI.

Finally, the research team is now experienced and prepared to conduct digital PPI workshops in the future. Even though digital tools and formats must be adapted with regard to workshop goals and participants, we now have a digital PPI toolkit available that allows us to conduct digital meetings more routinely and with less preparation.

### Creating an open, communicative and trustful atmosphere for digital PPI

As stressed by systematic reviews on PPI activities in general [23, 24], communicating with participants and building a personal relationship in advance proved crucial for effective PPI within our digital workshops as well. This was achieved from a distance by telephone contact, information packages, a technical introduction workshop and technical support via phone and by sending snacks and drinks for the workshop breaks to participants’ homes. In the evaluation interviews, participants told us that they felt supported and safe, as they trusted the research team. Most participants from all groups rated their chance to speak during the workshops as “very good”. They reported in evaluation interviews that they felt everyone was on equal footing and a facilitated change of perspectives was stressed as a major personal benefit by many participants. The PPI-associated benefits of empowerment and feeling valued, mutual trust and knowledge exchange [23, 25] could be achieved within a digital format as well.

Similar to Lampa et al. [6], we divided the overall workshop group in smaller groups during the SWSs to increase everyone’s chance to speak and encourage also rather cautious participants to chime in. This seemed reasonable as observation protocols showed that less interaction occurred between participants in the whole group during SWSs. To be responsive to power dynamics between physicians and HCAs/nurses as well as between health professionals and patients, we started with homogeneous stakeholder groups in the IWS to foster peer support and confidence before entering the discussion with all stakeholders in the SWS. Furthermore, participants appreciated the transparent and on-going documentation on the digital whiteboard that gave them a feeling of co-production and made the development process of the intervention visible and debatable. The wish of some participants for a meeting at which the final intervention is presented can hardly be put into practice, because the intervention is to be tested in a RCT against usual care. To prevent a contamination of the control group by providing them with information on checklist items and information channels between inpatient and outpatient settings, we cannot present the final intervention in detail at this point. However, the intervention development process and the rough results of this process have been compiled in a plain language brochure together with workshop participants, and the brochure will be distributed among all participants.

### Usability, technical support and technical skills of workshop participants

Similar to other experiences with and recommendations for conducting digital PPI [5, 6, 16], technical support and a technical introduction workshop proved crucial to break up barriers, enhance access and provide participants with a feeling of safety in our digital PPI workshops. Results from observation protocols showed that the major communication challenges were due to technical problems on the patients’ and health care professionals’ side, which were most often solved by technical support via phone or offering of alternative communication channels by the moderators. In the evaluation interviews participants from the patient group stressed that due to the technical introduction workshop, joining the digital meeting was possible without difficulty even for technically unexperienced individuals. In the chat feedbacks after each workshop, participants from the patient group assessed that they got along with the digital format and video conference system “very well” across all workshops, whereas the median rating in the health professional group for some workshops was only “good” with maximum ratings ranging up to “poor”. Remarkably, we did not offer the technical introduction workshop to health care professionals, because we had assumed that they were used to digital tools during their daily work. Furthermore, similar to findings from the HRA [1], we underestimated patients’ ability to use digital communication tools, whereas some of them told us in evaluation interviews that they used digital meeting systems for educational and leisure purposes. As a conclusion from these results, we will offer a technical introduction to all participant groups next time.

### Future directions for digital PPI (beyond the pandemic)

Similar to other studies [3–6], participants stressed the advantages of digital PPI workshops such as saving time, the possibility of combining workshop participation with family duties and getting to know patients and colleagues from across the country. Some participants actively wished to maintain digital formats also beyond the pandemic, especially because they felt that a sharing of perspectives and communication on equal footing was also possible in a digital format, and technical barriers were diminished by the technical introduction workshop and technical support during the meeting. For the research team the advantages of transregional collaboration in the organization and hosting of digital workshops such as a pooling of resources across teams also support the maintaining of digital workshop formats beyond the pandemic. Finally, the positive experiences of the HYPERION-TransCare workshops and especially patients’ positive feedback on the feasibility of digital PPI formats encouraged us to start our PBRN’s patient advisory board [13]—that we had postponed before—in a digital format as well. Some of the HYPERION-TransCare workshop participants were so enthusiastic about digital PPI that they decided to join our patient advisory board for long-term collaboration as well and even encouraged researchers to stick to a digital format, because this made them feel safe with regard to infectious risks.

Nevertheless, the positive attitudes and experiences of patients and health professionals in our workshop series cannot be generalized. During the recruitment process some participants declined participation because of the digital format of the participatory workshops. Furthermore, one participant dropped out after the technical introduction workshop, because he was not able to turn on his camera. Therefore, our workshop sample represents a positive selection of participants that feel comfortable with digital formats, even though some of them had minor experiences before. Like often with PPI, there is no “one size fits all” answer to the question of in-person vs. digital formats. Being in touch with patients and other stakeholders on that question and staying flexible and prepared with regard to both formats is the best research teams can do.

## Conclusions

In times of preventive reduction of physical contacts, digital formats using different didactic and documentation techniques, accompanied by technical support, can support active patient and public involvement and facilitate a change of perspective and mutual understanding between participants of different stakeholder groups across geographical distance. The advantages of digital formats such as geographical flexibility and saving time for participants as well as the opportunity to prepare and hold workshops in geographically stretched research teams persist beyond the pandemic and may in some cases outweigh the advantages of in-person communication.

## Supplementary Information

**Additional file 1**: Interview Guide for telephone interviews with participants from the HYPERION-TransCare digital PPI workshop series.

## Data Availability

The datasets generated and analyzed during the current study are not publicly available, because qualitative data (evaluation interviews and workshop protocols) cannot be anonymized totally due to context and content and we guaranteed participants not to pass on data to third parties. Anonymized data of participants’ feedback on workshops via chat is included in the published article.

## Abbreviations

BBB: BigBlueButton
HCA: Health Care Assistants
HRA: National Health Services Health Research Authority United Kingdom
IWS: Intensive Workshop
PBRN: Practice-Based Research Network
PPI: Patient and Public Involvement
SWS: Synthesis Workshop
