# Supplementary material for: Digital participatory workshops with patients and health professionals to develop an intervention for the management of polypharmacy: results from a mixed-methods evaluation and methodological conclusions
Source: Res Involv Engagem. 2022 Sep 16;8:52. doi: 10.1186/s40900-022-00387-1 (PMC9482272; doi:10.1186/s40900-022-00387-1)
Supplement: Supplementary file 1 — Additional file 1: Interview Guide for telephone interviews with participants from the HYPERION-TransCare digital PPI workshop series. [file 40900_2022_387_MOESM1_ESM.pdf]

Interview Guide for telephone interviews

with participants from the HYPERION-TransCare digital PPI workshop series

Thank you for your time and effort to give us feedback on our workshop series within HYPERION-TransCare. We aim to continue collaborating with patients, physicians, health care assistants, nurses and other stakeholders in the development of projects in the future. Therefore, we are very interested in how you experienced the workshops and in your suggestions for improvement.

1. First of all I would like to know: what was your motivation to participate in the HYPERION-TransCare workshops?

2. Did you have any concerns in advance? Which concerns? And how did they compare to the actual workshops?

3 [You participated in several workshops.] How did you experience the collaboration between the different stakeholder groups and professions?

- Was there something especially challenging?
- Did you experience inconvenient situations? Which?
- Is there something that you want to stress that you experienced as especially positive?

4. What is your impression: were you able to influence the development of the intervention? In what way? What was your most important contribution?

5. What is your advice for us for the planning of a similar project in the future?

- Based on your experiences within HYPERION-TransCare: would you participate again in a similar project? Which preconditions are mandatory in this case?

6. How did you like the digital format of the workshops?
